# Clinical Relevance of Mallampati Grading in Predicting Difficult Intubation in the Era of Various New Clinical Predictors

**DOI:** 10.7759/cureus.16396

**Published:** 2021-07-14

**Authors:** Mamta Harjai, Sharif Alam, Priyesh Bhaskar

**Affiliations:** 1 Department of Anesthesiology and Critical Care Medicine, Dr. Ram Manohar Lohia Institute of Medical Sciences, Lucknow, IND; 2 Department of Anaesthesiology, Career Institute of Medical Sciences and Hospital, Lucknow, IND

**Keywords:** airway, cormack-lehane grade, endotracheal intubation, general anesthesia, laryngoscopy

## Abstract

Background and aims

Management of difficult airway can be associated with serious morbidity and mortality and it is a basic and serious concern for anesthesiologists. The preoperative airway assessment is done by using conventional clinical predictors. The present study was conducted to find the correlation of various new clinical predictors with the Cormack-Lehane (CL) grade at the laryngoscopic view in patients undergoing general anesthesia with endotracheal intubation.

Settings and design

The prospective, comparative, observational, double-blind study was carried at Dr. Ram Manohar Lohia Institute of Medical Sciences, Lucknow.

Materials and methods

The study was conducted in 150 patients undergoing elective surgery under general anaesthesia. The primary outcome was the measurement of clinical airway assessment preoperatively based on certain parameters (inter incisor gap (IIG), modified Mallampati grading (MPG), neck circumference/thyromental distance (NC/TMD), ratio of height to thyromental distance (RHTMD)). The secondary outcome was the correlation of clinical airway assessment with CL grading to predict difficult intubation. The sensitivity, specificity, positive predictive value (PPV), and negative predictive values (NPV) of the parameters were assessed.

Statistical analysis

The association between different predictors and difficult laryngoscopy was evaluated using binary univariate logistic regression and multivariate logistic regression and the significant clinical predictors were assessed by using Pearson’s correlation. A p-value of < 0.05 was considered significant.

Results

The incidence of difficult intubation in this study was 13.3%. Among the clinical predictors, the Mallampati grading has the maximum receiver operating characteristic (ROC) and area under the curve (AUC) with 86.7 % sensitivity to predict difficult laryngoscopy followed by NC/TMD and body mass index.

Conclusion

Modified Mallampati grading still holds its significant value among new predictors in the assessment of difficult laryngoscopy.

## Introduction

The first major responsibility for the anesthesiologist is to provide adequate ventilation and oxygenation by securing the patient’s airway. Preoperative assessment of the patient’s airway facilitates the anesthesiologists to predict the ease of visualizing the glottis and to perform intubation easily. Various studies have shown the incidence of difficult intubation ranging between 9% and 16%[1,2] while the incidence of difficult intubation in the intensive care unit (ICU) or Emergency Medicine Department is probably as high as 20% [3]. Though there are several conventional clinical airway assessment parameters such as the modified Mallampati classification [4,5], hyomental distance, thyromental distance, neck movements, inter incisor gap, BMI, and ability to flex and extend the cervical spine, neck circumference, upper lip bite test, etc. which are usually used to predict a difficult airway preoperatively [6], the diagnostic accuracy of these predictors in predicting difficult intubation is low during pre-anesthetic airway assessment [7] and unexpected difficult intubations continue to occur. The diagnostic accuracy of these pre-anesthetic airway screening tests has varied from study to study [8]. Recently new clinical predictors like neck circumference/thyromental distance (NC/TMD), ratio of height to thyromental distance (RHTMD) have been studied in detail [9,10]. The primary aim of our study was to evaluate sensitivity, specificity, positive predictive value (PPV), negative predictive value (NPV), odds ratio (OR), and receiver operating characteristic (ROC) for various conventional clinical screening tests such as Mallampati grade (MPG), inter incisor gap (IIG), Mentohyoid distance, thyromental distance (TMD), neck circumference (NC), NC/TMD, RHTMD, to determine a precise as well as simple and clinically relevant parameter among conventional and new ones for predicting difficult laryngoscopy for regular practice in anesthesia to reduce the airway-related morbidity and mortality.

## Materials and methods

After getting approval from Ethical Committee (IEC no.77/17, CTRI/2019/02/017508), Dr .Ram Manohar Lohia Institute of Medical Sciences, Lucknow, UP, this prospective, comparative, observational and double-blind study was conducted. The study included 150 patients of ASA grade 1 and 2, aged 18-65 years, either sex without any known obvious airway pathology undergoing elective surgery under general anesthesia for 12-15 months. Informed consent was taken from all the patients. Patients who refused to give consent or patients with any feature of the obvious difficult airway due to facial anomalies, obesity (body mass index [BMI] >35 kg/m^2^), limited mouth opening and patients requiring rapid sequence intubation, with cervical spine pathology, uncooperative patients and pregnant patients were excluded from the study.

All patients underwent a detailed preoperative airway evaluation on the day before surgery by the anesthesiologist who was blinded to the study. The modified MPG, IIG, mentohyoid distance, TMD, NC, BMI, NC/TMD, RHTMD were noted and recorded for all patients.

Modified MPG [11] was done to assess the oropharyngeal view by asking the patient to sit and open his/ her mouth maximally and to protrude the tongue without phonation and record the structures visible upon maximal mouth opening and classified as score. Score 1 is when soft palate, fauces, uvula, and tonsillar pillars clearly visible, Score 2 is soft palate, fauces, and the uvula visible, Score 3 is soft palate and base of the uvula visible and Score 4 is when soft palate not visible.

IIG was assessed by measuring the space between the upper and lower incisors at the midline asking each patient to open the mouth to the maximum possible. The normal distance is typically >3.5 cm [12].

Mentohyoid distance was measured as the distance between the hyoid bone and the tip of the chin when the head was fully extended and the mouth closed. The normal distance is >5.3 cm.

TMD was measured as a distance from the thyroid notch to the mentum while the head was fully extended and mouth closed, employing a rigid ruler. The distance was rounded to the closest 0.5 cm and graded as, Class I: >6.5 cm, Class II: 6-6.5 cm and Class III: <6 cm. NC was measured at the level of the cricoid cartilage. The NC/TMD and RHTMD [13] were then calculated.

After preoperative assessments, on the day of surgery, the patient was shifted to the operating room with intravenous access. All standard monitors like ECG, noninvasive blood pressure (NIBP), pulse oximetry, temperature probe were connected and values noted. After preoxygenation of three to five minutes, IV midazolam (0.05-0.15 mg/kg) and IV fentanyl (2 μg/kg) were administered as premedication. The patients were induced by IV induction agent, IV propofol (2 mg/kg). After three minutes of administration of IV muscle relaxant Inj rocuronium (0.6-0.8 mg/kg), all patients were intubated by the senior anesthesiologist who had the experience of more than three years and was blinded to the findings of preoperative airway assessment. Direct laryngoscopy was performed using Macintosh blade. Glottic visualization was assessed and Cormack-Lehane (CL) grade was noted as Grade I when full glottic exposure was seen, while it was Grade II when only posterior commissure of glottis was seen, Grade III was only epiglottis visible and Grade IV when epiglottis was not even visible.

Intubation was classified as easy Laryngoscopy (CL Grade 1 and 2) and difficult laryngoscopy (CL Grade 3 and 4) characterised by poor glottic visualization. To preclude the bias in the results due to demand characteristics or the placebo effect, double-blind procedure was utilized in the study.

Statistical analysis

Statistical analysis was performed using commercially available computer software IBM SPSS software version 21 for windows IBM Corp., Armonk, NY, USA. Sample size was calculated on the assumption of incidence of difficult intubation as 10% [1,2] as seen in previous studies. Type 1 error was set at 4%. 138 patients provide a power of >80% to predict co-relation between the CL grading and the clinical predictors with a type I error of 3%. We enrolled 150 patients. Patient data were presented as mean ± standard deviation.

## Results

Total of 150 patients were recruited into our study, which included 64 men (42.7%) and 86 women (57.3%), with age ranging from 18 to 65 years. The incidence of poor visualization of larynx during laryngoscopy in our study was 13.3% (20 patients) and incidence of good visualization of larynx was 86.7% (130 patients). There were no patients in the extremely difficult group (CL Grade IV). Table 1 shows the distribution of the patients according to the CL grade at direct laryngoscopy. 77 patients had a CL Grade I (51.3%), 53 patients had a CL Grade II (35.3%), 20 patients had a CL Grade III (13.3%), and none of the patients had a CL Grade IV.

**Table 1:** Data demonstrating Cormack–Lehane (CL) score of patients.

CL grade	Visualization	Frequency	Percent
1.0	Full glottic exposure	77	51.3
2.0	Only posterior commissure of glottis seen	53	35.3
3.0	Only epiglottis visible	20	13.3
4.0	Epiglottis not visible	None	0
Total		150	100.0

The demographic profile, including age, sex, weight, height and BMI of easy and difficult laryngoscopy are shown in Table 2. Difference in age and height of patients of both groups were not found to be statistically significant, the male and female ratio was comparable in both easy and difficult laryngoscopy groups, while the difference in weight and BMI were found to be statistically significant (p = 0.005) when both groups were compared. Mean BMI of patients in easy laryngoscopy group was 24.31 ± 3.8 kg/mt2 and 27.42 ± 4.72 kg/mt2 in difficult laryngoscopy group.

**Table 2:** Demographic data of the easy and difficult laryngoscopy groups. ^1^Binary univariate logistic regression. *Significant p value (p < 0.05). CL: Cormack-Lehane.

Parameters	Easy (CL 1-2) (n = 130)		Difficult (CL 3-4) (n = 20)		^1^p-value
	Mean	±SD	Mean	±SD	
Age (year)	43.75	12.24	47.90	10.61	0.154
Sex: Male	57	43.85%	7	35%	
Female	73	56.15%	13	65%	0.720
Weight (kg)	61.46	11.31	67.30	7.67	0.027^*^
Height (cm)	158.72	8.10	157.65	10.38	0.599
BMI (kg/m^2^)	24.31	3.80	27.015	4.72	0.005^*^

The American Society of Anesthesiologists (ASA) grade I and II were comparable in both easy and difficult laryngoscopy. Clinical airway predictors like MPG, IIG, mentohyoid distance, TMD, NC, NC/TMD, RHTMD, were measured to anticipate laryngoscopy based on Cormack and Lehane's view as shown in Table 3. Out of 150 patients, 37 had MPG score I (28.46%), 76 (58.46%) with score II, 17 (13.08%) were found to have score III in easy group whereas 1 (5%) patient had MPG score I, 12 (60%) with score II, 4 (20%) with score III and 3 (15%) patients had score IV in difficult group. The MPG grade III and IV were found to be a strong predictor in anticipating difficult airway (p < 0.001), while the difference in inter incisor gap of patients of easy and difficult laryngoscopy group was found to be statistically significant (p < 0.001). The difference in mentohyoid distance, TMD, NC, NC/TMD and RHTMD of patients of easy and difficult laryngoscopy was not found to be statistically significant (p < 0.378 and p <0.096, p = 0.223, p = 0.062 and p = 0.253, respectively). Mean NC/TMD in patients of easy and difficult group was 3.802 ± 0.53 cm and 4.04 ± 0.44 cm, respectively.

**Table 3:** Clinical Parameters of the easy and difficult laryngoscopy groups according to binary univariate logistic regression analysis. ^1^Binary univariate logistic regression. *Significant p value (p < 0.05). CL: Cormack-Lehane; ASA: American Society of Anesthesiologists.

Parameters	Easy (CL 1-2) (n = 130)		Difficult (CL 3-4) (n = 20)		^1^p-value
	N	%	N	%	
ASA I	50	38.46	7	35	0.752
ASA II	80	61.54	13	65	
MPG Score 1	37	28.46	1	5	
2	76	58.46	12	60	
3	17	13.08	4	20	<0.001^*^
4	0	0.00	3	15	<0.001^*^
	Mean	±SD	Mean	±SD	
Inter incisor gap	4.80	0.65	4.08	0.90	<0.001^*^
Mentohyoid distance	5.36	0.77	5.20	0.69	0.378
Thyromental distance (TMD)	9.43	1.08	9.01	0.61	0.096
Neck circumference (NC)	35.43	3.90	36.55	3.02	0.223
NC/TMD	3.80	0.53	4.04	0.44	0.062
Ratio of height to thyromental distance (RHTMD)	17.07	1.82	17.54	1.30	0.253

The binary multivariate logistic regression was also used for analysis in BMI (kg/m^2^), MPG, IIG, mentohyoid distance, TMD, NC, NC/TMD, RHTMD, to determine the risk factors for difficult laryngoscopy as shown in Table 4. The IIG, TMD, NC and NC/TMD were found to be a significant independent risk factor for laryngoscopy.

**Table 4:** Binary multivariate logistic regression (forward-Wald) analysis performed in each patient group to determine the independent risk factors for difficult laryngoscopy in each population.

Variables	β	SE	Wald	p-value	OR	95% CI	
						Lower	Upper
BMI (kg/m^2^)	0.158	0.160	0.973	0.324	1.171	0.855	1.604
Mallampati grade	0.755	0.783	0.931	0.335	2.128	0.459	9.866
Inter incisor gap	-2.317	0.819	7.995	0.005^*^	0.099	0.020	0.491
Mentohyoid distance	-1.131	0.794	2.032	0.154	0.323	0.068	1.528
Thyromental distance (TMD)	-18.266	6.487	7.928	0.005^*^	-	-	0.004
Neck circumference (NC)	4.737	1.666	8.089	0.004^*^	114.139	4.361	2987.00
NC/TMD	-35.90	13.219	7.378	0.007^*^	-	-	-
Ratio of height to thyromental distance (RHTMD)	-0.936	0.603	2.407	0.121	0.392	0.120	01.279

Based on the ROC curve, the area under the curve (AUC) of BMI, MPG, IIG, mentohyoid distance, TMD, NC, NC/TMD and RHTDM were 0.670, 0.690, 0.268, 0.439, 0.376, 0.602, 0.679 and 0.592, respectively as shown in Table 5.

**Table 5:** Receiver operating characteristic (ROC) analysis of difficult laryngoscopy components and BMI, Mallampati grade, inter incisor gap, mentohyoid distance, thyromental distance (TMD), neck circumference (NC), NC/TMD and RHTMD.

	Area	SE	p-value	95% CI	
				Lower bound	Upper bound
BMI (kg/m^2^)	0.670	0.063	0.014	0.5477	0. 93
Mallampati grade	0.690	0.062	0.006	0.568	0.812
Inter incisor gap	0.268	0.061	0.001	0.149	0.388
Mentohyoid distance	0.439	0.075	0.379	0.293	0.585
Thyromental distance (TMD)	0.376	0.058	0.075	0.263	0.490
Neck circumference (NC)	0.602	0.058	0.144	0.488	0.715
NC/TMD	0.679	0.054	0.010	0.573	0.785
Ratio of height to thyromental distance (RHTMD)	0.592	0.059	0.185	0.476	0.708

In this test, maximum AUC was found in MPG followed by NC/TMD and BMI. The AUC for the modified Mallampati class was 0.690 among the clinical predictors indicating that it has the highest validity among the parameters studied. The sensitivity, specificity, NPV and PPV were used to analyze the Mallampati grade, Inter incisor gap, Mentohyoid distance, (Table 6). These tests were done to demonstrate the accuracy of risk factors. The cut-off points for difficult laryngoscopy were the Mallampati score≥3, Inter incisor gap <3.8, and Mentohyoid distance <5.26. Among these clinical predictors, the inter incisor gap was most sensitive (sensitivity of 89.3) and most specific (specificity of 50%) in predicting difficult laryngoscopy but PPV was 96.2% and NPV 25% as compared to Mallampati score which showed PPV and NPV as 100%.

**Table 6:** Sensitivity, specificity, positive predictive value (PPV), and negative predictive value (NPV) of the conventional clinical parameters in predicting a difficult laryngoscopy.

Test	Sensitivity	Specificity	PPV	NPV
Mallampati Score	86.7%	13.3%	100.0%	100.0%
Inter incisor gap	89.3%	50.0%	96.2%	25.0%
Mentohyoid distance	83.0%	4.5%	67.7%	10.0%

## Discussion

Difficult airway is the major factor of mortality and morbidity following anesthesia and is generally encountered during unexpected clinical conditions [14]. Though the use of video laryngoscopic devices and supraglottic airway devices has increased, direct laryngoscopy is still most frequently used as the first device for intubation. It remains a challenge to date to identify the patients in whom laryngoscopy and intubation may be difficult. Several studies in different clinical fields have been conducted to identify predictors of difficult laryngoscopy and intubation [15-17].

Demographic data (age and sex distribution) was comparable between easy and difficult laryngoscopy during intubation. Among clinical predictors for difficult airway assessment, the weight of patients showed a significant difference in easy and difficult laryngoscopy (p = 0.027). BMI (kg/m^2^) was also associated with difficult airway (p = 0.005). This finding was supported by another study by Helene Gonzalez et al [18] who found that difficult tracheal intubation was more frequent in obese than in lean patients (14.3% vs 3%; p = 0.03). Increase in fat distribution specifically in the anterior neck in patients of high BMI might be a relevant predictor of difficult intubation in this subset of patients [18].

The MPG grade (III & IV) was found to be highly statistically significant (p < 0.001) in the present study for the easy and difficult intubation. The Mallampati test, when considered as a single predictor for difficult laryngoscopy had a sensitivity of 86.7%. Previous studies which have used Mallampati as a single predictor had a wide range of sensitivity from 40% to 82.4% [6,11,19] which could be attributed to the inter-observer variability for assessing MPG grading. In our study, the tests were carried out by the same investigator, thereby avoiding inter-observer variability.

In our study, another clinical predictor that was found to be statistically significant (p < 0.001) was the inter incisor gap. Wilson et al. [12] in their study showed that the inter incisor gap was significantly smaller in those patients where laryngoscopy was difficult. Our findings were in accordance with another study done by Abdel Raouf et al. [20] who concluded that inter incisor gap <4 cm was associated with difficult laryngoscopy. Insertion of laryngoscope or airway devices in patients with less inter incisor gap obscures the vision of the larynx. 

The other parameters like mentohyoid distance, TMD, NC were not found to be significant independent predictors. In the present study, the mento hyoid distance was measured in neutral position in spite of measuring in the position in which laryngoscopic intubation is actually performed. The diagnostic value of TMD was found to be unsatisfactory as an individual predictor in Meta-analysis performed by Shiga et al. [17]. It has been observed in many previous studies [21], that neck circumference of obese patients was found to have significant effects on difficult intubation at 40 cm of neck circumference, the probability of difficult intubation was approximately 5%, and at 60 cm of neck circumference, the probability of difficult intubation was approximately 35% and concluded that increase in neck circumference was associated with difficult intubation in obese patients [22]. The disparity in the present study and study done by others can be explained by the exclusion criteria of the present study which did not include the patients with BMI>35 kg/mt2. The Neck Circumference is not a perfect indicator alone because the amount of soft tissue at the various region of neck is different [23]. But when multivariate binary logistic regression analysis was used, we found that neck circumference was statistically significant to differentiate between easy and difficult laryngoscopy with p= 0.004.

The difference in NC/TMD of patients of easy and difficult laryngoscopy was not found to be statistically significant (p = 0.062) during univariate logistic regression analysis. This is because, in the present study, we did not found NC or TMD as a significant predictor and patients with a value of NC/TMD >5 cm was seen in only 6 patients out of 150. But on using binary multivariate logistic regression analysis (Table 4) to determine the independent risk factors for difficult laryngoscopy, we get NC/TMD as a statistically significant (p=0.007) factor. Sangeeta D et al. [23], Yadav et al. [24] and Rajani et al. [25] also found NC/TMD as a significant predictor in their study.

In the present study, RHTMD was not found to be statistically significant (p = 0.253). Our study found the maximum value of RHTMD as 22.67. The result was not in accordance with the various studies like Schmitt et al [26] (cut off of RHTMD>25) and Krobbuaban et al [27] (cut off of RHTMD>23.5) who concluded that RHTMD was a good predictor for difficult laryngoscopy. Azim et al [10] also found high sensitivity with RHTMD (64.7%) and interpreted it to be a simple preoperative test for screening. In another Indian study [28], authors demonstrated that the RHTMD can also be used as an acceptable alternative for better airway predictability other than ULBT which can be used as a simple bedside screening tool for prediction of difficulty during intubation, but it should always be combined with other airway assessment tests.

The IIG has been demonstrated to be one of the most sensitive single predictors of a difficult airway in normal patients [12,20,29]. This is in accordance to our study where the maximum sensitivity and specificity was observed in IIG while in another recent study done by Yong-Zheng Han et al. [30] the ratio of NC and IIG (RNIIG) was found to be a new and simple predictor with a higher level of efficacy, and they suggested that it could help in better management of difficult laryngoscopy specially in patients with cervical spondylosis.

ROC curve was obtained for various clinical variables. ROC Curve is a statistical graph to represent the sensitivity and specificity of any test and the AUC is an effective measure for assessing the inherent validity of the test and it will help to know to what extent the test is diagnostic. Diagnostic accuracy is directly proportional to the area under the curve. In our study, the AUC was found maximum in Mallampati grade (0.690) followed by NC/TMD and BMI indicating that these may be used for bedside screening tests for determination of difficult laryngoscopy. Hence, MPG with 86.7 % sensitivity, maximum ROC and maximum AUC can be a good predictor of difficult laryngoscopy. By assessing and comparing the results obtained from all the above-mentioned clinical tests, we found that each one has its advantages and disadvantages and all variables showed large indecisive results that may explain previous inconsistent results in the prediction of difficult laryngoscopy so combination of these predictors rather than single one can be more helpful in anticipation of difficult intubation beforehand.

Limitations of study

The sample size of our study may be small and a larger study is warranted for confirmation of the findings. Secondly, there were some extra variables that were difficult to control like position used during intubation, number of attempts at laryngoscopy and intubation, the view of glottis used during Cormack-Lehane’s grading (though we tried to minimize this by including the best view as the first view or the view just before passing the endotracheal tube) . However, we took all precautions to minimize these factors. We tried to decrease inter-observer variability bias by keeping the same person for observation of clinical parameters.

## Conclusions

In conclusion, we found that several airway assessment parameters IIG, TMD and NC/TMD were statistically significant independent risk factors for difficult laryngoscopy. But MPG along with inter incisor gap still holds its significant value as a single, simple bedside predictor among new predictors in assessment of difficult airway. Combining it with other single valuable predictors will increase the probability of correct diagnosis. Further studies with tests which may involve combination of two or more simple bedside predictors to device a simple airway assessment tool are warranted for best clinical results.
